# Intra and inter‐network connectivity in the olfactory network and default mode network across the Alzheimer's disease spectrum: An analysis of COMPASS‐ND cognitive and imaging data

**DOI:** 10.1002/alz70856_098697

**Published:** 2025-12-24

**Authors:** Tristin Best

**Affiliations:** ^1^ Concordia University, Montreal, QC, Canada

## Abstract

**Background:**

Olfactory dysfunction is a prevalent and early symptom of Alzheimer's disease (AD), often preceding the core memory impairment features of AD. Moreover, brain regions integral to olfactory processing are implicated in the early AD pathophysiology. Previous work has identified olfactory deficits in individuals with subjective cognitive decline (SCD), the earliest at‐risk stage of AD. Few studies, however, have examined the associations between cognitive and olfactory changes, and their neural correlates, in this population.

**Method:**

Data from the Comprehensive Assessment of Neurodegeneration and Dementia (COMPASS‐ND) were used, including resting‐state functional MRI and neuropsychological data. Participants included individuals with SCD (*n* = 128), mild cognitive impairment (MCI; *n* = 315), AD (*n* = 116), and cognitively unimpaired older adults (*n* = 84) who served as controls. We examined group differences and associations between olfactory dysfunction, as measured by the Brief Smell Identification Test, and cognition. Independent components analysis was used to identify the resting‐state olfactory network (ON) and default mode network (DMN). We examined intra‐ and internetwork functional connectivity in the ON and DMN and related these findings to olfactory performance and cognition.

**Result:**

We observed groupwise deficits in olfactory and cognitive function in MCI and AD groups relative to controls (*p* < 0.01), but not between SCD and controls (*p* > 0.05). Poorer olfactory performance in the MCI and SCD groups was related to worse memory (*p* < 0.05) but not in controls (*p* > 0.05). We observed increased connectivity within the ON in SCD and decreased connectivity in MCI and AD, relative to controls (*p*‐fdr < 0.05). We expect to find alterations in connectivity within the DMN and between the ON and DMN, which we expect to relate to poorer olfaction and cognitive performance.

**Conclusion:**

These findings suggest a coupling of olfaction and memory performance in SCD, that is not present in controls, despite similar sensory and cognitive performance. Moreover, we expect these changes will relate to changes in connectivity within and between the ON and DMN. These data contribute to the potential utility of measuring olfactory performance as a non‐invasive and reliable biomarker in SCD and MCI for conversion to later stages of the AD continuum.

